# Homo-binding character of LMO2 isoforms and their both synergic and antagonistic functions in regulating hematopoietic-related target genes

**DOI:** 10.1186/1423-0127-17-22

**Published:** 2010-03-27

**Authors:** Wei Sun, Wen-Wen Shen, Shuang Yang, Fen Hu, Yang Gao, Yu-Huan Qiao, Tian-Hui Zhu

**Affiliations:** 1 Laboratory of Molecular Genetics, College of Medicine, Nankai University, Tianjin 300071, PR China

## Abstract

**Background:**

The human *lmo2 *gene plays important roles in hematopoiesis and is associated with acute T lymphocyte leukemia. The gene encodes two protein isoforms, a longer form LMO2-L and a shorter form LMO2-S. Both isoforms function as bridge molecules to assemble their partners together to regulate their target genes. A typical LMO2 binding site consists of two elements, a GATA site and an E-box, with an interval of 9~12 bp.

**Methods:**

In this study, the combination of MBP pulldown assay and mammalian two hybrid assay were used to confirm the homo-binding character of LMO2-L/-S isoforms. Luciferase reporter assay and Real-time PCR assay were used to detect expression levels and relative promoter activities of LMO2-L/-S isoforms. Co-transfection and Luciferase reporter assay were used to reveal the detailed regulatory pattern of LMO2-L/-S isoforms on their targets.

**Results:**

Herein we report the homo-interaction character of LMO2-L and LMO2-S and their major difference in manner of regulating their target genes. Our results showed that LMO2-L and LMO2-S could only bind to themselves but not each other. It was also demonstrated that LMO2-L could either positively or negatively regulate the transcription of its different target genes, depending on the arrangement and strand location of the two elements GATA site and E-box, LMO2-S, however, performed constitutively transcriptional inhibiting function on all target genes.

**Conclusion:**

These results suggest that LMO2 isoforms have independent functions while there is no interaction between each other and they could play synergetic or antagonistic roles precisely in regulating their different genes involved in normal and aberrant hematopoiesis.

## Background

LMO2 is a pivotal factor in promoting embryonic hematopoiesis as well as angiogenesis[1,2]. Lmo2 null mutation in mice leads to failure of yolk sac erythropoiesis and embryonic lethality around E10.5[1]. LMO2 also correlates to the onset of T cell leukemia[3-5]. The human lmo2 gene was firstly cloned from an acute T lymphocyte leukemia (T-ALL) patient with (11;14)(p13;q11) translocation[6], and its aberrant expression could be detected in a considerable percentage of T-ALLs[5]. The oncogenic property of LMO2 was also confirmed in transgenic mouse models[7] and in X-SCID patients treated with retrovirus-mediated gene therapy, in which T-ALL emerged due to the insertion of retroviral sequence that incurs aberrant expression of LMO2 [8].

**Figure 1:**
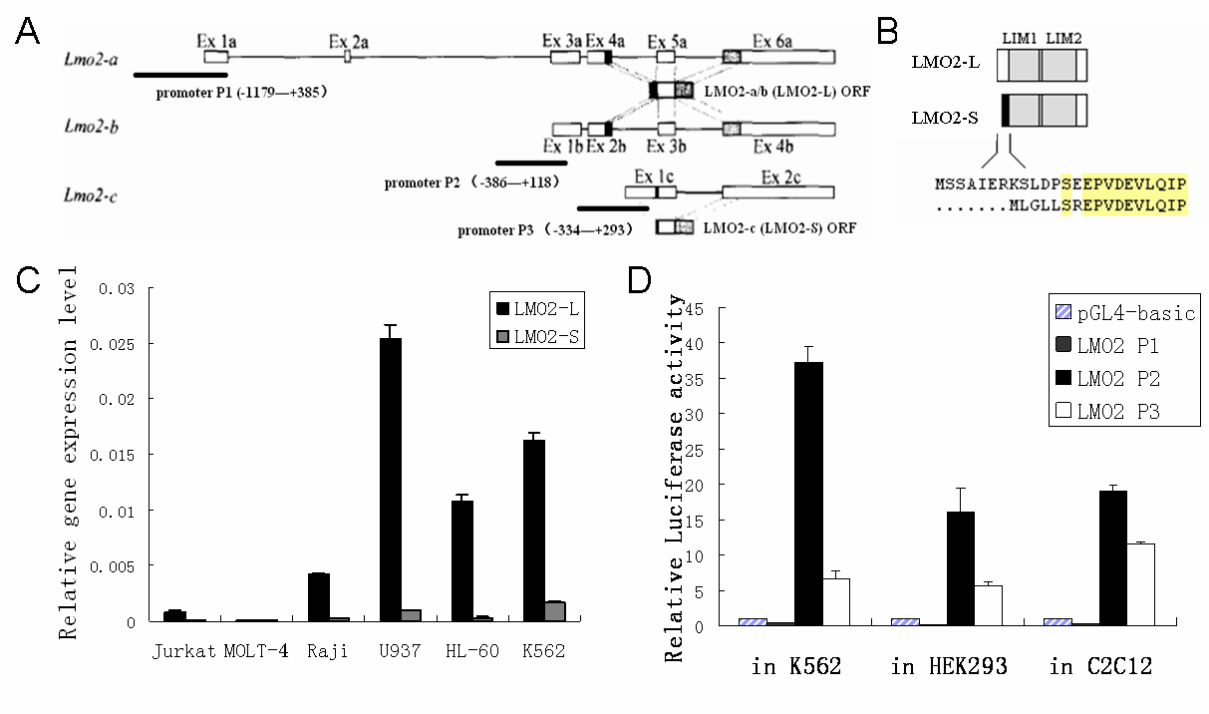
**Expression of LMO2-L and -S isoform and activities of their promoters in haematopoietic and non-haematopoietic cells**. (A) Sketch map of three transcripts of LMO2. *Lmo2*-a/b which have 6 and 4 exons respectively encode LMO2-L, and *Lmo2*-c that has 2 exons encodes LMO2-S. The 3 black bars represent 3 promoters P1, P2 and P3, the regions used for reporter assays were marked below. (B) Difference in protein sequences between LMO2-L/S. The cartoon shows the difference in N-terminal regions and the different amino-acids are shown beneath. (C) Real-time PCR detection of LMO2-L/S expression in cell lines. The bars represented the ratio between relative expression levels of LMO2-L/S and GAPDH, the data showed the means of three separate experiments. (D) Relative activity of P1, P2 and P3. pGL4-basic was used as control and its activity was marked as 1. All the data came from at least three independent experiments and were evaluated by student's *t*-test, p < 0.05.

Early studies showed that *lmo2 *gene had two transcripts *Lmo2-a *and *Lmo2-b *with distinct promoters, but both of them encode a same product LMO2-L[9]. Our group later cloned a new transcript from human adult kidney, termed *Lmo2-c *(Fig. 1A)[10]. *Lmo2-c *has the promoter of its own and encodes a shorter isoform, termed LMO2-S, which has 14 amino acids missed in the N-terminal region compared to LMO2-L (Fig. 1B).

Both LMO2-L and -S are transcriptional regulators, but interestingly, they have no direct DNA binding ability. They function as bridge molecules to assemble their partners, including LDB1, GATA1, TAL1 and E47, together to form a complex that that recognizes and binds to specific DNA sequences of the target genes. The specific DNA sequences of LMO2 binding site consist of a GATA site and an E-box, with 9~12 bp in between[11,12]. Till now several LMO2 targets have been identified, including *c-kit*[13], *GPA*[14] and *miR-142*[15] in hematopoietic cells and *VE-Cadherin*[16] in vessel endothelial cells. It is also suggested that the binding site in T-ALL cells could be double E-boxes[17]. Both LMO2-L and LMO2-S can interact with all these partners including LDB1, GATA1, TAL1 and E47 in a similar manner, but the binding affinity can vary for LDB1[18]. It remains uncharacterized in detail regarding how the two LMO2 isoforms function together in a given cell type. In this study, we report that both LMO2-L and LMO2-S had only homo-interaction patterns, and they could have either synergic or antagonistic transcriptional regulatory roles depending on their target genes.

## Methods

### Plasmid construct

**Figure 2:**
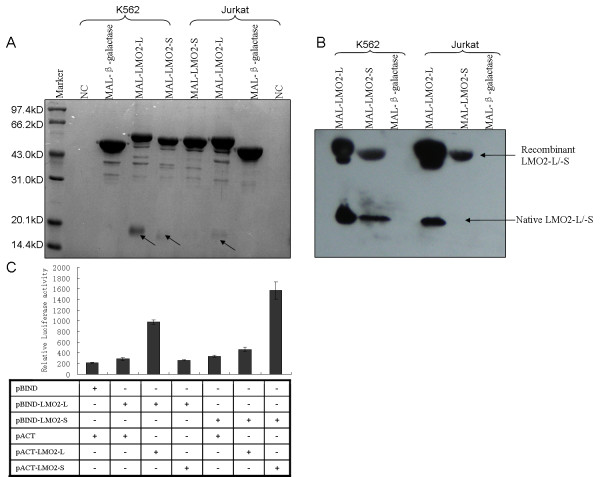
**MBP-pulldown and mammalian two hybrid assay indicated homo-interaction character of LMO2-L and -S isoform**. (A) Coomassie blue R-250 staining of MBP-pulldown samples after SDS-PAGE. NC, amylose resin without recombinant protein and incubated with K562/Jurkat total protein; MAL-β-galactase, sample of purified MAL-β-galactase incubated with K562/Jurkat total protein used as a control; MAL-LMO2-L/-S, sample of purified MAL-LMO2-L/-S incubated with K562/Jurkat total protein. (B) Western blot analysis of relative samples corresponding to (A) using anti-LMO2 antibody. (C) Mammalian two hybrid assay of LMO2-L and LMO2-S. pACT, activation domain fusion protein expression plasmid; pBIND, GAL4 binding domain fusion protein expression plasmid. +, cells transfected with such component; -, cells transfected without such component.

The promoter region of *Lmo2-a*, *-b *and *-c *transcripts termed P1, P2 and P3 were cloned from normal human genome and inserted into pGL4-basic vector. The artificial reporters were generated as follows: DNA sequences containing the typical LMO2 binding sites, the E-box and GATA site in different arrangements were synthesized (Fig. 2B), the sequences were: E-box-GATA+: 5'-GATCTACAGGTGCTATGCGGGGATAGA-3';

E-box-GATA-: 3'-ATGTCCACGATACGCCCCTATCTCTAG-5;

GATA-E-box+: 5'-GATCTGGATAGCTATGCGACAGGTGA-3';

GATA-E-box-: 3'-ACCTATCGATACGCT GTCCACTCTAG-5;

E-box-E-box+: 5'-GATCTACATCTGCTATGCGGACAGATGA-3';

E-box-E-box-: 3'-ATGTAGACGATACGCCTGTCTACTCTAG-5.

DNA fragments in pairs were annealed in vitro using T4 DNA ligase buffer (Takara, Dalian, China). The annealing temperature fell in a gradient of 1°C/min from 95°C to 25°C. Then the double-strand fragments were inserted into the BglII site just ahead of the basic SV40 promoter in the pGL3-promoter vector.

### Cell culture and transfection

K562, U937, HL-60, Jurkat, MOLT-4 and Raji cells were cultured in RPMI1640, HEK293 cells were cultured in DMEM, and both media were supplemented with 10% fetal bovine serum (GIBCO BRL, Grand Island, NY, USA), 100 μg/ml penicillin and 100 μg/ml streptomycin. Two millions of K562 cells were transfected by electroporation with a total of 10 μg plasmid DNA and electroporation was performed with 960 μF and 210 V in a Bio-Rad gene-pulser^®^. HEK293 cells were plated at 1 × 10^5 ^cells/well in 24-well plates and transfected by Lipofectamine2000 following the manufacturer's instruction (Invitrogen, Austin, TX, USA).

### Induced expression of MBP-LMO2-L/-S recombinant protein and maltose affinity chromatography

DH5α strains with plasmid pMAL-c2x/pMAL-LMO2-L/-S were activated and cultured in 1 L flask, IPTG (at a final concentration of 0.1 mmol/L) was added at a culture density of OD 0.6-0.8 to induce the expression of recombinant protein for 4 hr. Bacteria cells were harvested and re-suspended in STE buffer (50 mmol/L Tris-HCl pH 7.9, 0.5 mmol/L EDTA, 50 mmol/L NaCl, 5%Glycerol) in a ratio of 1: 10 with the original media. Bacteria protein was extracted by sonication (250 W, 10 s, 5 times). 10 mg total bacteria protein of each sample was firstly incubated with 5 mL amylose-resin (New England Biolabs, Ipswich, MA, USA) at 4°C for 2 hrs with rotation. Then the protein-resin mixture was filled in the 1 × 10 cm column (Phamacia), washed with 5 × volume Buffer A (20 mmol/L Tris-HCl pH 7.9, 0.2 mol/L NaCl, 1 mmol/L EDTA) and eluted by Buffer B (Buffer A with 20% maltose). Elutions were dialyzed in 4°C for 1 hr with 3 times in Buffer C (20 mmol/L Tris-HCl pH 7.9, 0.1 mol/L NaCl, 1 mmol/L CaCl_2_, 1 mmol/L ZnSO_4_), concentrated by air-dry and run SDS-PAGE.

### Factor Xa cleavage of MBP-LMO2-L/-S recombinant protein

Purified recombinant MBP-LMO2-L/-S proteins (1 mg each) in Buffer C were added 5 μg Factor Xa (New England Biolabs), respectively and incubated in 4°C for 72 hrs. Then samples were run SDS-PAGE or native PAGE.

### MBP-pulldown assay

Total K562 or Jurkat proteins were extracted using mRIPA lysis buffer (50 mmol/L Tris-HCl pH 7.5, 150 mmol/L NaCl, 1% Triton X-100, 0.5% DOC, 10 ug/mL Aprotinin, 10 ug/mL Leupeptin, 1 mmol/L PMSF). 0.3 mg of purified protein was firstly incubated with 1 mg total K562/Jurkat protein at 4°C overnight. Then 100 μL of amylose-resin (New England Biolabs) was added into each sample and incubated at 4°C for 2 hrs with rotation. Incubated resin of each sample was washed 3 times with Buffer A. Then equal volume 2× SDS loading buffer was added to the resin precipitate and the samples were heat-denatured for 10 min. The samples were then run SDS-PAGE, gel was stained by Coomassie blue R-250.

### Western blot analysis

The same sample derived from MBP-pulldown assay was used. After SDS-PAGE, proteins were transferred to a nitrocellulose membrane, and detected using anti-LMO2 monoclonal antibody (Millipore, Billerica, MA, USA). Immunostaining was detected using an enhanced chemiluminescence system (Amersham Pharmacia Biotech, Buckinghamshire, UK).

### RNA isolation and Real-time PCR

Total RNA was isolated from cells using Trizol reagent (Invitrogen) and 0.5 μg of each sample was used for cDNA synthesis by M-MLV (Promega, Madison, WI, USA). Real-time PCR was performed by ABI PRISM 7000 (ABI, USA), using 1× Evagreen dye and ROX as passive reference. The amplification parameters were: 95°C 3 min, followed by 95°C 30 s, 64°C 1 min, 40 cycles. Primers used for detection are:

LMO2-L forward: 5'-CGAAAGGAAGAGCCTGGAC-3';

LMO2-S forward: 5'-CGGTGCTGGTCTCACTCTG-3';

LMO2-L/S reverse: 5'-TTCACCCGCATTGTCATCT-3';

miR-142 forward: 5'-TCTTAGGAAGCCACAAGGAG-3';

miR-142 reverse: 5'-TAAGGTGCTCACCTGTCACA-3';

GPA forward: 5'-ATTGTCAGCAATTGTGAGCATA-3';

GPA reverse: 5'-TGATCACTTGTCTCTGGATTTT-3';

c-kit forward: 5'-GTGAAGTGGATGGCACCTGA-3';

c-kit reverse: 5'-TTGATCCGCACAGAATGGTC-3'.

The relative gene expression levels were normalized by housekeeping gene GAPDH.

### Luciferase assay

The ratio of Luciferase reporter to Renilla was 9:1 in a total of 10 μg for electroporation of K562 cells. For transfection of HEK293 and C2C12 cells with Lipofectamine 2000, the ratio was 5:1 in a total of 2 μg, and 4 μl of Lipofectamine 2000 were mixed for each well of 24-well plate. In the experiments of co-transfection of both reporters and trans-regulator vectors, each single trans-regulator vector was mixed with the reporter in a ratio of 1:1 and the ratio between Luciferase reporter and Renilla was 5:1. The total plasmid was 2 μg, and 4 μl of Lipofectamine 2000 were mixed for each well of 24-well plate. In the experiments of co-transfection with all members of LMO2 complex, in which either LMO2-L or LMO2-S or no LMO2 (LMO2-NULL) was involved, the ratio of Ldb1:LMO2:TAL1:E47:GATA1:reporter was 2:2:1:1:1:1. In the case of E-box-E-box, the ratio of Ldb1:LMO2:TAL1:E47:reporter was 2:2:2:2:1, according to the previously established models. The ratio between Luciferase reporter and Renilla was 5:1. The total plasmid was 2 μg, and 4 μl of Lipofectamine 2000 were mixed for each well of 24-well plate. Cells were lysated 24 hrs after transfection. Luciferase activity was measured using Dual-Luciferase Reporter Assay Kit (Promega) and normalized by Renilla luciferase activity, according to the manufacturer's instruction.

### Mammalian two hybrid assay

LMO2-L and -S coding sequence was cloned into pACT and pBIND vector (Promega), respectively. The pACT-LMO2-L/S, pBIND-LMO2-L/S and pG5luc reporter vector were co-transfected into HEK293 cells in a ratio of 1: 1: 1 by Lipofectamine 2000. Luciferase activity was measured using Dual-Luciferase Reporter Assay Kit (Promega) following the manufacturer's instruction 24 hrs after transfection.

## Results

### Cell type-specific expression level and the promoter activities of LMO2 isoforms

The mRNA expression level of LMO2-L (corresponding to *Lmo2-a/b*) and LMO2-S (corresponding to *Lmo2-c*) were firstly detected by real-time PCR in several hematopoietic cell lines (Fig. 1C). In T lineage cells Jurkat and MOLT-4, there were almost neither LMO2-L nor LMO2-S expression detected. In B lineage cell line Raji, the expression level of LMO2-L was modest while LMO2-S was hardly detectable. However, in all three myeloid cell lines, U937, HL-60 and K562, the expression of both LMO2-L and -S was apparent, with LMO2-S always lower than LMO2-L.

Furthermore, the activity of *LMO2-a*, *b *and *c *promoters, termed P1, P2 and P3, respectively, was measured by their capability to drive luciferase expression in human hematopoietic cell line K562, human non-hematopoietic cell line HEK29,3 and non human cell line C2C12, respectively (Fig. 1D). The promoter region from -3433 to +385 for P1 was cloned and a series of 5'- and 3'- truncated forms were generated. Surprisingly, none of these fragments showed any positive activity in any cell lines (data not shown). P2 activity, however, was about 8 fold higher than P3 in K562 cells. This well matched the real-time PCR results that LMO2-L was expressed always at the higher level than LMO2-S (Fig. 1C). In both HEK293 and C2C12 cells, a basal level of both P2 and P3 activities was observed, though P3 again appeared weaker than P2. This result implicated that P2 and P3 have certain basal activities in mammalian cells and the two promoters are stringently regulated in hematopoietic cells.

### Homo-interaction character of LMO2 isoforms

**Figure 3:**
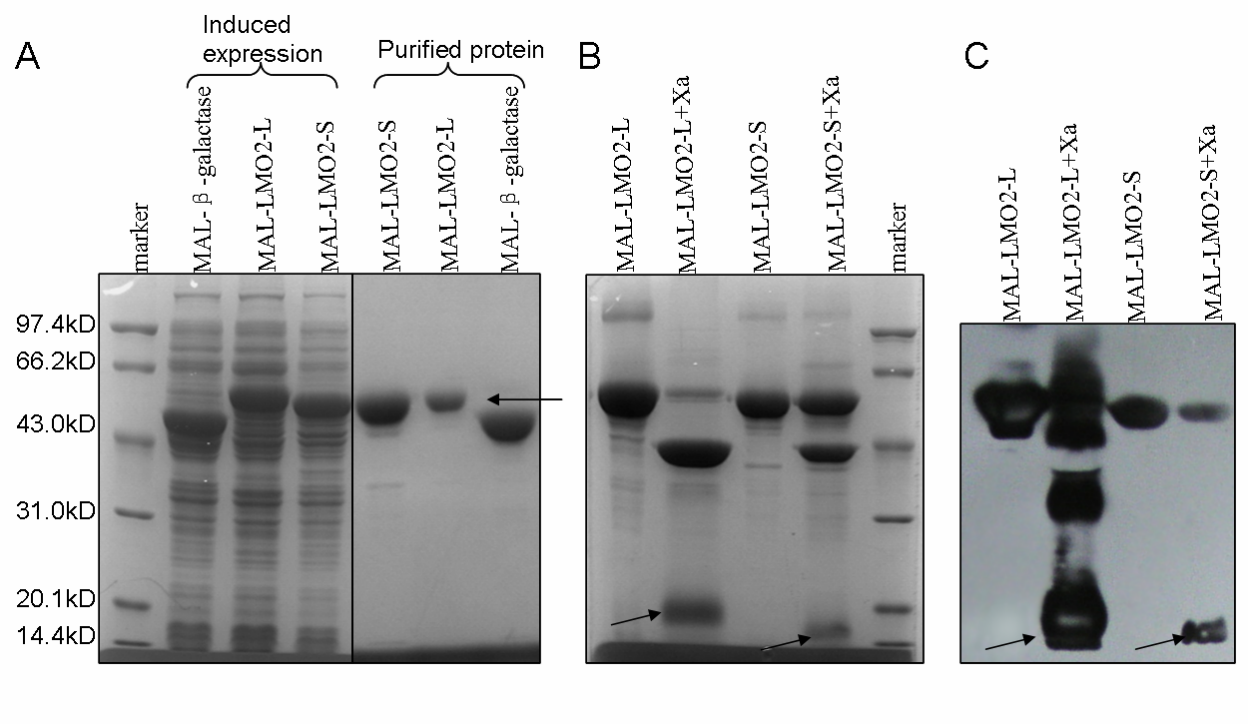
**Maltose affinity chromatography purification of MBP-fusion proteins and Factor Xa cleavage**. (A) Coomassie blue R-250 staining of purified MBP-LMO2-L/-S/-β-galactase samples after SDS-PAGE. (B) Coomassie blue staining of Factor Xa cleaved MBP-LMO2-L/-S samples after SDS-PAGE. (C) Western blot analysis of relative samples corresponding to (B) using anti-LMO2 antibody.

The maltose-binding protein (MBP) tagged recombinant LMO2-L/S fusion proteins, which can be expressed in E. coli in soluble form were purified by maltose affinity chromatography with amylose resin, SDS-PAGE showed the MBP-LMO2 fusion proteins were in a high purity (Fig. 3A). Purified MBP-LMO2-L or -S recombinant proteins were then cleaved by Factor Xa according to the peptide sequence between MBP and LMO2-L/-S. LMO2-L/-S bands were visible on SDS-PAGE with Coomassie blue staining (Fig. 3B) and both cleaved and fusion LMO2-L/-S protein could be detected by Western blot using anti-LMO2 antibody as arrows indicated (Fig. 3C).

In the MBP-pulldown assay, purified MBP-LMO2-L or -S recombinant proteins were incubated with K562 or Jurkat cell lysate overnight with rotation to enrich the components that can bind with LMO2-L or -S, respectively. Meanwhile, the resin bound with MBP or without bacteria proteins were used as controls. After washing to eliminate unbound component, the proteins bound to the resin were denatured and run SDS-PAGE, the gel was stained by Coomassie blue (Fig. 2A). The clear 60 kD bands in the gel were the MBP-LMO2-L/S fusion proteins, these were subsequently confirmed by Western blot analysis (Fig. 2B). Notably, there were also two visible bands in K562 lysate lanes as indicated by the arrows, which were approximately 18 kD and 17 kD, respectively. These two bands were quite likely to native LMO2-L and -S in K562 cells no matter the molecular weight or the relative expression amount, and these were also confirmed by Western blot subsequently (Fig. 2B). Interestingly, Jurkat cell was considered had no LMO2 expression, but in this assay, after MBP-pulldown enrichment, trace LMO2-L could also be detected while LMO2-S could not (Fig. 2B).

In the other hand, in mammalian two hybrid assay, in cells that cotransfected with pACT-LMO2-L/pBIND-LMO2-L vector or pACT-LMO2-S/pBIND-LMO2-S vector, the Luciferase activity was high, however, in other cases (pACT-LMO2-L/pBIND-LMO2-S or pACT-LMO2-S/pBIND-LMO2-L), the activity was low (Fig. 2C). This result indicated that the interaction between two molecules of LMO2-L or two molecules of LMO2-S were strong, and the interaction between each other was weak.

### Different regulation modes by LMO2-L and -S isoforms on various promoter element arrangements of target genes

**Figure 4:**
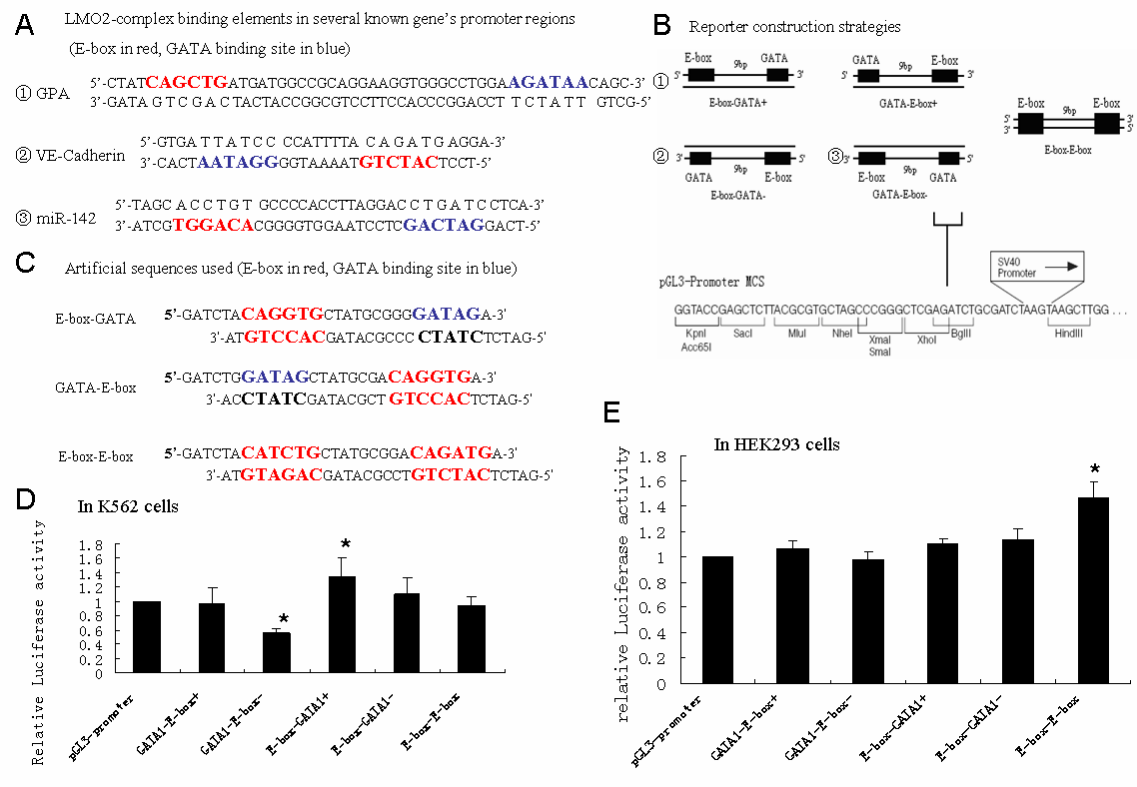
**Regulation activities of LMO2 isoforms on five element arrangements within a typical LMO2 binding site**. (A) Sequences of promoter regions of GPA, VE-Cadherin and miR-142. Letters bigger and in red were indicated as E-box, and in blue as GATA site. (B) Sketch map of the reporter construction strategies. The two black boxes as well as their commentaries indicated 5 different arrangement forms of LMO2 binding site. (C) The artificial sequences used were shown in the table. Letters bigger and in red were indicated as E-box, and in blue as GATA site. (D) Relative activity of each artificial reporter in K562 cells. (E) Relative activity of each artificial reporter in HEK293 cells. For both (D) and (E), the activity of pGL3-promoter was used as control and marked as 1. All these experiments were performed at least 5 times independently, the graph showed the means and the standard error of means of each result. All the data were evaluated by mean square test and Dunnett-*t *test, *Dunnett-*t *test, p < 0.05.

The LMO2 binding site consisting of a GATA site and an E-box was asymmetric as it was not a palindrome for the whole site. It has been notable that GATA1 and TAL1/E47 bind to their relevant DNA binding sites as two arms of the LMO2 complex, which make the whole complex asymmetric. So far, three different arrangement forms of LMO2 binding elements have been identified in the promoter region of *GPA*, *VE-Cadherin *and *miR-142*, respectively, as shown in Fig. 4A. In these genes, LMO2-L has different regulatory functions, to inhibit the expression of miR-142[15] and up-regulate the other two in the presence of its partner molecules [14,16]. Therefore, it could be speculated that arrangements of LMO2 binding elements may affect the regulation pattern of the LMO2 complex on these targets. To confirm this, 5 different artificial reporter constructs were made in this study (Fig. 4). These constructs were named based on the strand localization of the sense sequence of the GATA site, as the sequence of E-box was a palindrome (Fig. 4B).

In K562 cells that have endogenous expression of both LMO2 isoforms and their co-factors, the activity of some reporters was shown obviously different (Fig. 4D): the activity of GATA-E-box- showed a dramatic decrease compared to the control while the activity of E-box-GATA+ showed a modest increase. However, in HEK293 cells which had been shown to have no expression of GATA1, TAL1 or LMO2-L/S (data not shown), the activity of these reporter constructs was indistinctive except the construct E-box-E-box, which was shown to be slightly increased (Fig. 4E).

**Figure 5:**
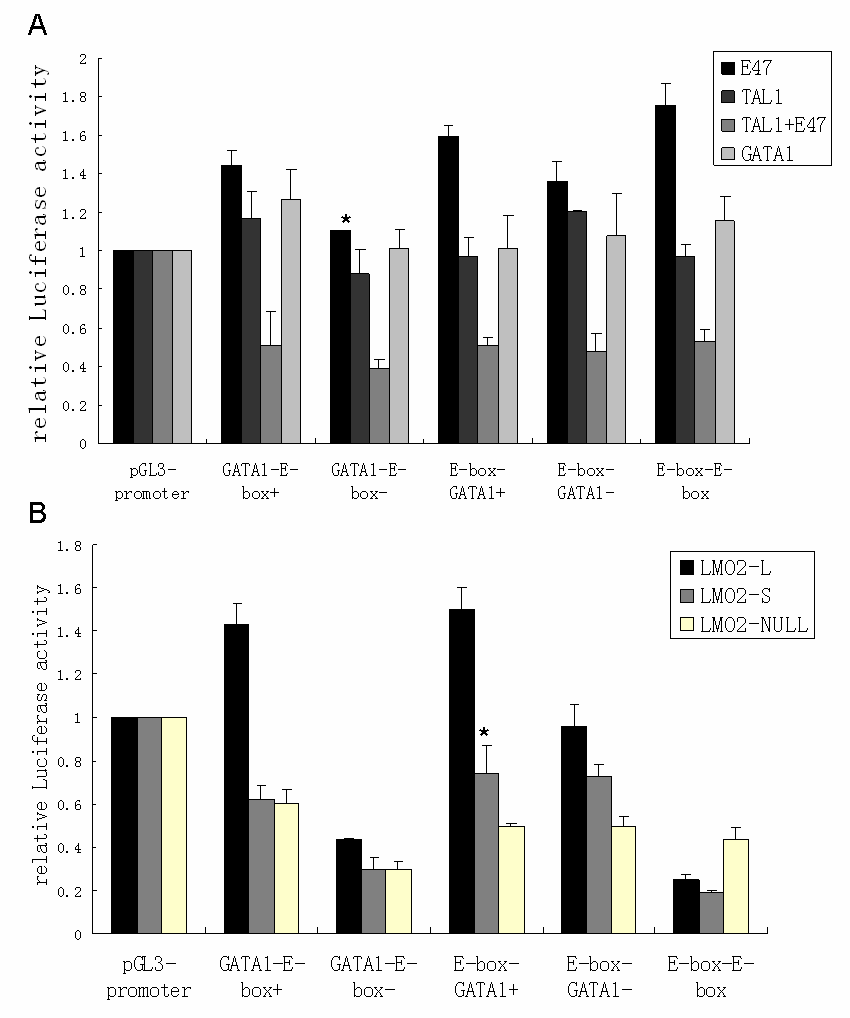
**Relative activities of reporter constructs regulated by LMO2 isoforms in HEK293 cells**. (A) Relative activity of each artificial reporter upon co-transfection of E47, TAL1, E47 and TAL1, or GATA1. (B) Relative promoter activity of each artificial reporter upon co-transfection of all other members of LMO2-complex with LMO2-L, LMO2-S, or without LMO2 (LMO2-NULL). For both (A) and (B), the activity of pGL3-promoter was used as control and marked as 1 in each group and all the others were shown as the ratio to it. All these experiments were performed at least 5 times independently, the graph showed the means and the standard error of means of each result. All the data were evaluated by mean square test and Dunnett-*t *test, *Dunnett-*t *test, p < 0.05.

The regulatory roles of *trans*-regulators involved in LMO2 complex as well as two LMO2 isoforms on different *cis*-element reporters were further investigated. The function of three members that could direct bind to DNA, including GATA1, TAL1 and E47 were firstly tested (Fig. 5A). E47 could increase the activity of all 5 reporter constructs while TAL1 alone had little effect on any form of the reporters. However, in the co-presence of both E47 and TAL1, where the two molecules could form heterodimers, the activity of all 5 reporters was decreased down to 50% compared to the control. GATA1 alone had no prominent effect on all 5 reporters. It was remarkable that although these regulators, when alone or combined, had regulatory effects on the reporter constructs, their functions did not appear to be selective. The regulators tested in this study exhibited same functions on all these reporter constructs regardless the arrangement difference of LMO2 binding elements.

However, in the presence of LMO2-L, the performance of each reporter changed significantly; the activity of GATA-E-box+ and E-box-GATA+ was up-regulated whilst the activity of GATA-E-box- was down-regulated dramatically and no prominent activity change was detected for E-box-GATA-. The activity of E-box-E-box was also further inhibited in the presence of LMO2-L. In the presence of LMO2-S, however, the performance of these reporters appeared totally different. The LMO2-S complex always exhibited inhibitory function on all the reporter constructs, at least down to the level of the LMO2-NULL group. Particularly, the activity of E-box-E-box was further inhibited, lower than LMO2-NULL and similar to LMO2-L (Fig. 5B).

### Effects of LMO2-L and -S isoforms on regulating endogenous expression of target gene in myeloid cells

**Figure 6:**
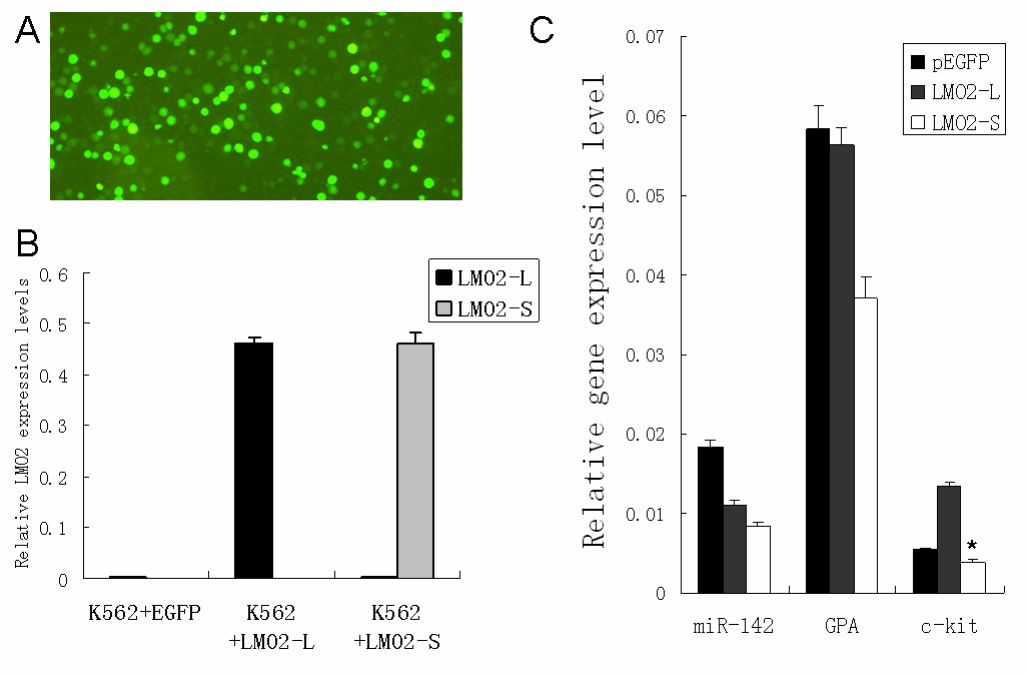
**Differential regulation of LMO2-L and -S isoforms on endogenous expression of their target genes in K562 cells**. (A) Transfection efficiency was monitored by the observation of EGFP fluorescence and estimated more than 80%. (B) Total expression levels of LMO2-L or LMO2-S in cells transfected with EGFP, LMO2-L or LMO2-S. (C) Relative expression levels of miR-142, GPA and c-kit in the same sample of (B). The bars in (B) and (C) represented the ratio between the expression levels of LMO2-L/S and housekeeping gene GAPDH. The expression levels in the cells transfected with EGFP were considered having no prominent changes and used as control. The data showed the means of three independent experiments. All the data were evaluated by student's *t*-test, *student's *t*-test, p < 0.05.

*GPA*, *c-kit *and *miR-142 *were all known target genes of the LMO2 complex and normally expressed in myeloid cells. Following the transfection with LMO2-L/S or the EGFP control, their endogenous expression level changes in K562 cells were also detected by real-time PCR. While the elecrtoporation efficiency reached about 80%, as monitored and estimated by the observation of EGFP expressing cells (Fig. 6A), transgenic expression of LMO2 L/S seemed to be predominate and the endogenous expression level of the two LMO2 isoforms were very low (Fig. 6B). Then the same cell samples were used to detect the relative expression levels of the three target genes *GPA*, *c-kit *and *miR-142 *(Fig. 6C). It was found that cells over-expressing LMO2-L had *miR-142 *level down-regulated and *c-kit *level increased, On the other hand, cells over-expressing LMO2-S had all the three genes decreased significantly.

## Discussion

LMO2 is a crucial factor in hematopoiesis as well as onset of T-ALL. It always functions as bridging molecule to assemble several *trans*-regulators to form a transcriptional regulatory complex and determines the regulation patterns on its targets. The homo-interaction character of LMO2 isoforms indicated that *trans*-regulators could be assembled by either LMO2-L or LMO2-S, but there should be in principle no LMO2-L/LMO2-S heterodimer in such complex. This seemed to facilitate different functions performed by LMO2-L and LMO2-S without interfere. Moreover, because the difference of amino-acid sequence between LMO2-L and LMO2-S is in their N-terminal, it could be speculated that the N-terminal of LMO2-L/-S was involved in their homo-interaction and was important for their functions *in vivo*.

There were several known targets of LMO2, including early-progenitor genes *c-kit*[13], *runx1*[19], erythrocyte specific gene *GPA*[14], *p4.2*[20] and T cell related gene *miR-142*[15] and *RALDH2*[21]. Interestingly, although these genes are regulated by the same LMO2 complex, formed by either LMO2-L or LMO2-S together with GATA, TAL1, and E47, some of them are shown to be positively regulated while others negatively [13-15]. Further analysis showed that the arrangement of the two key elements, GATA site and E-box, in the promoter region of these genes varies significantly. In this study, we demonstrated that the functional responses of these arrangements to the two LMO2 isoforms were indeed different. LMO2-L could enhance the activity of promoters with GATA-E-box+ or E-box-GATA+ while dramatically inhibiting that of the GATA-E-box- or E-box-E-box arrangement. However, LMO2-S exhibited the constitutively inhibitory functions on all the arrangements. Therefore, the eventual regulation outcomes by LMO2 isoforms on specific target genes in particular cell types apparently depend on the element arrangements in the promoter regions of these target genes as well as the relative expression level between the two LMO2 isforms.

Considering this precise regulation mechanism of LMO2-L/S on their targets, the stringent regulation on the expression of LMO2-L and LMO2-S would be crucial for normal cellular processes including differentiation and proliferation. In this respect, several previous studies have reported that expression regulation of LMO2-L and -S are indeed regulated by a series of mechanisms. There exists a negative regulatory element in *Lmo2-a *promoter (P1) [22] and the ETS-family members Fli1, Elf1, and Ets1 can up-regulate *Lmo2-b *promoter (P2) activity [23]. The *Lmo2-c *promoter (P3), on the other hand, can be enhanced by GATA1 and inhibited by another ETS-family member PU.1 [18]. As demonstrated in this study, LMO2-S was always expressed at lower levels compared to LMO2-L in hematopoiesis-related cells and the basic promoter activity of LMO2-L was about twice of LMO2-S. It is therefore inferable that in different lineages and different stages of hematopoiesis, the maintenance of the relative expression levels and consequently the finely-controlled functions of the two LMO2 isoforms may be accomplished by these multiple factors.

The E-box-E-box form of the LMO2 binding site was thought to be the aberrant LMO2 binding site particularly in T-ALL [12,17]. One explanation for such a model is that LMO2-L-TAL1 complex could inhibit E47-mediated transactivation [24]. There have been several examples for this regulatory manner, such as the regulation on artificial reporters of the *Cd4*, *preTα*, and *Tcrα/δ *genes [24-26]. Our results here showed a general transcription inhibiting manner for this case by both LMO2-L and LMO2-S. It could further explain the pathologic function of LMO2-L and -S in T-ALL as both of them could further inhibit the expression of its aberrant targets bearing such arrangement of LMO2 binding site. Although there are both synergetic and antagonistic functions between LMO2-L and -S, both of them could function as oncogene through the negative regulation on such genes in the case of aberrant expression and thus block normal cell differentiation and cause leukemia.

## Conclusion

Taken together, our results showed that LMO2-L and LMO2-S had only homo-binding character but not binding to each other. Meanwhile, LMO2-L could either positively or negatively regulate the transcription of its different target genes, depending on the arrangement and strand location of the two elements GATA site and E-box, LMO2-S, however, performed constitutively transcriptional inhibiting function on all target genes. These results suggest that LMO2 isoforms have independent functions while there is no interaction between each other. They could play synergetic or antagonistic roles precisely in regulating their different genes involved in normal and aberrant hematopoiesis.

## Competing interests

The authors declare that they have no competing interests.

## Authors' contributions

WS: performed the experiments and wrote the paper. WWS: performed most parts of the experiments. SY: help revising the paper. FH: performed parts of the experiments. YG: help revising the paper. YHQ: guide for some experiments. THZ: design the experiments and corresponding author.
